# Physiological and Behavior Monitoring Systems for Smart Healthcare Environments: A Review

**DOI:** 10.3390/s20082186

**Published:** 2020-04-12

**Authors:** Mariana Jacob Rodrigues, Octavian Postolache, Francisco Cercas

**Affiliations:** 1 Iscte–Instituto Universitário de Lisboa, Av. das Forças Armadas, 1649-026 Lisboa, Portugal; opostolache@lx.it.pt (O.P.); francisco.cercas@lx.it.pt (F.C.); 2 Instituto de Telecomunicações, Av. Rovisco Pais, 1, 1049-001 Lisboa, Portugal

**Keywords:** healthcare, internet of things, smart environments, physiological signs monitoring, activity recognition, indoor air quality

## Abstract

Healthcare optimization has become increasingly important in the current era, where numerous challenges are posed by population ageing phenomena and the demand for higher quality of the healthcare services. The implementation of Internet of Things (IoT) in the healthcare ecosystem has been one of the best solutions to address these challenges and therefore to prevent and diagnose possible health impairments in people. The remote monitoring of environmental parameters and how they can cause or mediate any disease, and the monitoring of human daily activities and physiological parameters are among the vast applications of IoT in healthcare, which has brought extensive attention of academia and industry. Assisted and smart tailored environments are possible with the implementation of such technologies that bring personal healthcare to any individual, while living in their preferred environments. In this paper we address several requirements for the development of such environments, namely the deployment of physiological signs monitoring systems, daily activity recognition techniques, as well as indoor air quality monitoring solutions. The machine learning methods that are most used in the literature for activity recognition and body motion analysis are also referred. Furthermore, the importance of physical and cognitive training of the elderly population through the implementation of exergames and immersive environments is also addressed.

## 1. Introduction

Healthcare systems combined with the Internet of Things (IoT) technology help improve quality of life, health and life expectancy. These systems can be effectively implemented by acquiring environmental data, as well as the subject’s behavioral and physiological information from various distributed smart sensors. Following the IoT concept, all collected data is securely sent to the cloud and can be processed by data analysis algorithms to ascertain the existence of actuation patterns and make the system react to unexpected changes. These smart sensors can be expressed by smart wearable objects that provide health status information in unobtrusive ways, by sensing units embedded on daily used objects (e.g., smart wheelchair) and by other sensing systems that monitor indoor environmental conditions, including noise levels, air quality and lighting quality. As one of the growing industries, IoT for healthcare has been widely explored by academia [1,2], with a special focus on providing personalized, secure and effective health monitoring of patients. Such monitoring becomes an essential element for the older population that, with the increasing average of life expectancy, may develop mental and physical illnesses.

The concept of smart home or smart environment is generally given to environments with smart technologies that provide to their inhabitants useful services that substantially help improve their life quality. Such services consist of common automated mechanisms and functions that give the ability to monitor and control home appliances, windows, air conditioning system, doors and so forth. However, there are a wide variety of services that classify smart homes differently and that provide different kinds of support and assistance to the users in their daily life activities and actions [3]. Assistive services, which are specifically tailored to the special needs of the user, are part of an assisted living environment. These services are mainly focused on acquiring high level information about the user by monitoring their physical condition, performed activities and indoor localization.

**Figure 1:**
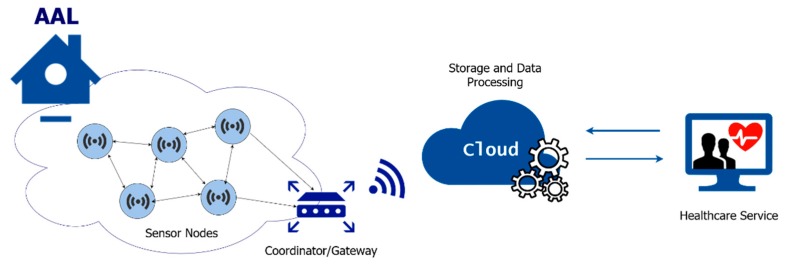
General architecture of a healthcare Internet of Things (IoT) system.

Ambient assisted living (AAL) is built on top of those smart home services and require the deployment of better monitoring technologies and assistive tools. AAL systems are specially designed to support the elderly and people affected by chronical diseases in their daily routine [4]. The main goal of AAL systems is to improve the life quality of the elderly and to extend the time they can live independently. These systems are expressed by an ecosystem of wearable and non-wearable medical sensors, wireless sensor and actuator networks (WSANs) and software applications that, when interconnected, provide a complete overview of the patient’s health status for specific environmental conditions, as well as a provision of the required healthcare services [5]. That is, an AAL system is based on the architecture of a healthcare focused IoT system. Figure 1 presents a general overview of such kind of systems, which are composed of environmental quality sensor nodes, actuator nodes and wearable devices. The information from the sensor network is sent to a coordinator node/gateway and then to cloud centers, where data is processed by data analysis algorithms.

Health-related data can be remotely obtained by any healthcare entity (caregivers, doctors and physiotherapists) while patients live independently in their preferred environment, due to telehealth and telemedicine concepts [6,7]. This is important because medical diagnostics taken directly from the patient’s home can greatly reduce hospital bills and allow a better treatment, specifically tailored for the patient. Real-time monitoring can be used to prevent many medical emergencies, such as heart failure, asthma attacks, among others. The option to live in their own home while benefiting of such services is a great necessity for the elderly, as verified by Cahill et al. [8]. The authors conducted a study to identify the requirements of elderly people and other stakeholders (i.e., nurse, family and career) when considering the deployment of an assisted living environment. They interviewed elderly that had independent lives, care nurses, family members and experts on dementia. Several requirements and needs were reported by the participants regarding resident experience: every participant expressed their will on staying at their private home, rather than going to a long-term care facility or residential homes. However, despite the comfort and privacy that private homes provide, the social activities and relationships that can be stimulated among the care community in nurse houses are much less than those possible at their homes. One way to overcome this problem is to consider the monitoring of the patients’ daily life, including activities associated with active aging (e.g., practice of physical exercise) and social interaction, and notify family members or caregivers when there might be social isolation or lack of mobility.

A variety of AAL based smart home projects have been reported in the literature for the last decade. Most projects provide efficient solutions to improve an individual’s independence in their living environment while monitoring the activities of daily living or physiological status in real-time. However, few consider the implementation of indoor environmental quality (IEQ) monitoring solutions, particularly of indoor air quality (IAQ) and indoor lighting quality (ILQ), which play a major role in human health and well-being. There is also an urgent need to stimulate cognitive and physical well-being for elderly people, which may develop mental illnesses and be more subjective to other negative events, such as social isolation and decline in mobility. These issues demand that AAL environments include personalized and effective solutions to enhance social engagement and to stimulate cognitive and physical activities in the elderly. 

This paper aims to address all the necessary components for the implementation of such a healthcare system, namely an AAL environment. This research focuses on discussing current AAL solutions presented in the literature, characterized by vital signs monitoring systems, and identifies the most relevant physiological parameters that need to be considered in order to provide viable health diagnostics. Indoor localization technologies for user location and daily activities’ recognition will be also addressed, as well as the most suitable machine learning and signal processing algorithms for activity recognition and pattern classification. Additionally, various monitoring solutions for indoor environmental quality assessment and cognitive and physical stimulation based on immersive environments, are included in this paper, respectively.

This paper is organized as follows: after this introductory section, Section 2 addresses physiological parameters monitoring systems and considers the most important vital signs that should be monitored in order to give an appropriate assessment of an individual’s health conditions; Section 3 refers to indoor tracking and localization technologies that are most suitable for daily activity recognition and body motion analysis; Section 4 focuses on the importance of indoor environmental quality and how its monitoring and control can be achieved; Section 5 comprises an analysis of the best machine learning algorithms that have been used in the literature for daily activity recognition; Section 6 addresses the importance to include exergames and immersive environments in a AAL system, as well as the current solutions found in the literature and Section 7 concludes the paper.

## 2. Health Status Monitoring Systems

The monitoring of physiological parameters and daily activities of patients is the main objective of healthcare services related to the implementation of assisted living environments. Wearable medical sensors play a critical role on such systems, as they collect health-related information that can be used to elaborate diagnostics in real-time of human health conditions [9]. As noted earlier, an AAL system may be based on medical sensors that, when connected to home gateways, send medical data to health monitoring systems in real-time. Wireless sensor networks (WSNs) are used to connect sensors to smart gateways and healthcare applications, thus allowing caregivers or physicians to monitor patients remotely.

In order to ascertain an individual’s health status and his reaction to external factors, it is necessary to monitor the various physiological parameters that are considered relevant. Over the years the following five vital signs have been examined: temperature, heart rate, blood pressure, respiratory rate and blood oxygen saturation [10]. These can be obtained through non-invasive and non-intrusive sensors, which can be included in long-term health monitoring systems [11]. Such sensors are mostly referred to as wearable sensors. They can monitor and record in real time information concerning an individual’s physiological condition and motor activities, without causing discomfort nor interrupting the practice of their daily activities. These wearable biomedical sensors measure physiological signs that can be used to obtain electrocardiograms (ECGs), electromyograms (EMGs), photoplethysmograms (PPGs), seismocardiograms (SCGs), ballistocardiograms (BCGs), blood pressure and body temperature and determine the heart rate (HR), oxygen saturation (SpO_2_), respiration rate (RR) and many other parameters. These sensors are generally connected in a wireless body area network (WBAN) or body sensor network (BSN), and can be placed directly on top of the skin, over clothes or even implanted in the person’s tissue. 

The next subsection considers the main vital signs that contribute for a better monitoring of human health and wellness, and the sensors and methods for acquiring and analyzing such parameters.

### 2.1. Cardiovascular Monitoring

There are several methods to record and monitor the cardiac activity using non-invasive techniques. The most widely used technique and diagnostic tool for healthcare environments is the electrocardiogram (ECG), which measures the electrical activity of the heart. An ECG is visualized by the formation of a waveform characterized by five peaks and valleys named P, Q, R, S, T and U, respectively, and each one reflects the physiological conditions of the patient’s heart and its main blood vessels. The QRS complex indicates ventricular depolarization and has a short duration if the heart is working efficiently. The R wave, or peak, is the first positive wave of the complex and it is used to determine the patient’s HR and heart rate variability (HRV), regarding the time period between its occurrences (called RR intervals) [12,13].

This method uses Ag-AgCl electrodes (wet electrodes) that must be affixed in specific areas of the body. However, the conducting gel that surrounds the electrode, and which serves as a conduction medium between the skin and the electrode, can produce irritant effects on the skin when used for longer periods. Another potential drawback associated with long-term use is surface degradation of electrodes, which leads to the deterioration of signal quality [14].

For this reason, ECG monitoring based on wet electrodes is less reliable when considering long term monitoring of the cardiac activity and it cannot be used without affecting the individual’s daily activities. Several alternatives for replacing these traditional electrodes have been suggested in the literature. Dry textile electrodes can be embedded in custom clothes, such as undershirts and bras, for ECG recording. This method proved to be usable for continuous ECG monitoring as stated by Tsukada et al. [15]. The characteristics of hydrophilic and flexible material of the hitoe^®^ textile electrode allowed it to easily adapt to the human’s skin surface. Due to the conductive polymers (PEDOT:PSS) being firmly anchored between nanofibers [16], the hitoe^®^ textile pad permits repetitive washings without impairing its electrical conductivity.

An and Stylios [17] designed textile electrodes that include a motion sensor with a textile-based electrode. This hybrid textile electrode was able to synchronously record ECG and motion signals. Both signals, when acquired simultaneously, are beneficial for the diagnosis of heart diseases. Changes in heart rate occur during or following a behavior such as changes in posture, walking and running. The association of daily physical activity derived from motion data with an ECG is very useful for cardiologists as it can help them determine the cause of a certain heart disease, e.g., abnormal ECG caused by over-exercising. Based on the numerous advantages of e-textile electrodes, many other researchers reported the use of such technology and materials for wearable ECG monitoring systems [18,19,20,21].

Smart textile systems based on fiber optic sensors have also shown to be a viable method to monitor respiratory and cardiac activity. The system proposed by Presti et al. [22] implements a smart textile based on a fiber Bragg grating (FBG) to detect small chest motions induced by the heart beating. By placing the smart textile at three different chest positions, the authors investigated the influence of measurement points in terms of signal amplitude and FBG performance, which is something that has not been considered in previous FBG-based studies.

As another alternative, the photoplethysmography (PPG) technique has proven to be a great alternative against the ECG [23,24], especially for HRV, HR and SpO_2_ measurements. It is considered a non-invasive and unobtrusive method. It uses a light source and a photodetector placed in contact with the skin’s surface to measure volumetric variations of blood circulation in veins and arteries [25]. Optical absorption or reflection of light is associated with the amount of blood that is present in the optical path. Changes in blood volume are synchronous with the heartbeat. A PPG sensor is usually placed at peripheral body sites, to measure the volumetric variations in the microvascular beds [26]. Parts of the human peripheral vascular system that can be used to place the sensor’s coverage area include the finger, earlobe and toe.

As an example, Malhi et al. [27] designed a wearable device based on a PPG sensor placed on the finger, within a wrist strap and a finger glove. This system gives users the possibility of free movement, without affecting their daily activities.

A wrist worn device, as designed by the authors in [28], includes a channel for cardiac activity monitoring based on photoplethysmography (PPG) and a body kinematics measurement channel for daily motor activities assessment, therefore enabling multiparametric monitoring in non-invasive and non-obtrusive ways.

Mary et al. [29] reported the development of a physiological parameter measurement system based on wearable devices to monitor human body temperature, heart rate and oxygen saturation using the PPG signal. The proposed system also used a three-lead ECG to determine if the patient whether had normal heart rate, or tachycardia or bradycardia conditions.

PPG also poses as a great solution for real-time and continuous detection of atrial fibrillation (AF), one of the most common types of arrhythmia. The detection of this cardiac rhythm disturbance can be based on the implementation of statistical analysis, machine learning and deep learning approaches. Pereira et al. [30] reviewed different studies based on these algorithms for AF detection through the evaluation of PPG signals. The authors highlight the main challenges that PPG-based AF detection comprises in clinical applications and how the different classification approaches address those limitations.

Additional unobtrusive techniques include ballistocardiography (BCG), which is used to measure repetitive motions of the human body, associated with cardiac cycles. It is one of the oldest non-invasive methods for cardiac–respiratory monitoring and can be used to get information about the activity of the heart, its condition and breathing patterns. Its graphical representation consists of the action–reaction force caused by the heartbeat and the pump of blood through the aorta [31]. The IJK wave complex from the BCG represents the ejection phase of the cardiac cycle. These main waves and time intervals between them reflect the physiological condition of the subject’s heart and its main blood vessels. BCG systems can either require mechanical connection between the subject’s body and the sensor or can be performed by contactless devices, which are, for example, ultrasonic sensors [32] and the microwave Doppler radar [33]. The main devices requiring mechanical contact are piezoelectric sensors, load cells, sternal accelerometers and electromechanical film sensors (EMFi). The robustness of BCG monitoring systems based on EMFi sensors has been evidenced in its large number of implementations [31,34,35,36]. However, the adoption of this solution in medical facilities is still limited in present days.

Innovative solutions that have been emerging in the literature have a new way to provide advanced and continuous physiological signal acquisition rely on the development of soft electronic circuits and highly stretchable systems. Electronic systems that can be attached to the epidermis allow a more comfortable and accurate measurement of human physiological conditions, when compared to the traditional systems. The physical properties of such devices offer levels of stretchability and thickness that are compliant to those of the skin, allowing a more precise and noninvasive mechanical connection with its surface, and therefore reduce motion artifacts and other limitations usually offered by common wearable systems.

Kim et al. [37] reported the development of multifunctional sensing platforms for temperature, strain and electrophysiological measurements, alongside other circuit elements based on ultrathin and elastic layouts. The authors were able to obtain ECGs, EMGs and electroencephalograms (EEGs) using the referred epidermal electronic system. Xu et al. [38] proposed a thin, soft stretchable electronic system for wireless electrophysiological monitoring. The designed device incorporates a microfluidic construction to allow elastic stretchability and flexibility and mechanically isolate rigid electronic materials. The device is wirelessly powered, incorporates radio frequency transmission capabilities and provides high precision measurement of ECGs, EMGs, electrooculograms (EOGs) and EEGs. This multifunctional solution allows long-term human health monitoring without constraining body movements and affecting the person’s daily activity.

Webb et al. [39] introduced an ultrathin, flexible and epidermal sensor technology for continuous monitoring of the microvascular and macrovascular blood flow. The sensor relies on thermal measurements of blood flow beneath a certain area of the skin. Comparisons to commercial optical blood flow measurement systems, and under immobilization of the subjects, validated the measurement accuracy of the reported system.

Another innovative solution based on a stretchable and lightweight wearable device was presented by Ha et al. [40]. An electronic tattoo (e-tattoo) was developed for both ECG and SCG measurement. Previous SCG are still based on rigid and non-stretchable materials, which was revealed to be uncomfortable to wear, especially in long-term use. In this work, the authors rely on a piezoelectric polymer, polyvinylidene fluoride (PVDF), to construct a stretchable vibration sensor capable of acquiring SCG signals. The synchronous collection of data from both ECG and SCG techniques increases the system’s efficiency on determining cardiac health conditions. This simultaneous acquisition of SCG allowed the validation of ECG readings accuracy, as well as the extraction of cardiac time intervals, which can be used for continuous estimation of blood pressure. The novel characteristics of these new wearable technologies are very promising for the future implementation of healthcare monitoring systems related to ambient assisted living.

Besides acquiring information about cardiovascular status, these methods can also be used to measure the autonomic nervous system (ANS) response and the person’s emotional state. This is done through HRV analysis, which is based on the study of the variation of the time interval between consecutive heart beats (RR intervals or peak to peak intervals). This analysis can quantify the sympathetic and parasympathetic nervous system to understand the overall status of the ANS. Its clinical importance includes the possibility of predicting mortality after the occurrence of an acute myocardial infarction, diabetic neuropathy and other neurologic disorders [12,13]. Both branches of the ANS are involved in the regulation of HR, with the sympathetic activity having a tendency of increasing the HR and decreasing HRV, whereas the parasympathetic activity decreasing the HR and increasing HRV [41]. HRV can be evaluated by three different methods [12,13]: time-domain, frequency-domain and non-linear methods. The simplest to implement is the time domain measurement, in which the time interval between successive heart beats is determined. The most common time-domain variables for statistical measurements include the mean RR interval, mean HR, the difference between the shortest and longest NN interval (where NN corresponds to time intervals between normal pulse peaks), standard deviation of the NN intervals (SDNN), root mean square of successive NN interval differences (RMSSD), standard deviation of successive NN interval differences (SDSD) and the number of successive intervals differing more than 50 ms (NN_50_). Frequency domain methods are better to discriminate between sympathetic and parasympathetic activities of the HRV. The power spectrum density (PSD) is estimated, in most cases, using a fast Fourier transform (FFT) and provides basic information about the distribution of power (i.e., variance) over frequency. For short term recording periods, whose standard is 5 minutes, three spectral components are measured [12]: the very low frequency (VLF, ≤ 0.04 Hz), low frequency (LF, 0.04–0.15 Hz) and high frequency (HF, 0.15–0.4 Hz). Finally, non-linear methods are used to analyze HRV. The most common measures are the Poincaré plot, approximate entropy (ApEn), sample entropy (SampEn), detrended fluctuation (DFA), correlation dimension and recurrence plots [13]. The time-domain, frequency-domain and nonlinear methods for HRV analysis are summarized in Table 1.

### 2.2. Body Temperature Monitoring

Body temperature is an important parameter for determining an individual’s general health condition and is considered one of the key vital signs. It can estimate the degree of “sickness” of an individual by studying the difference of body temperature obtained with the one that is considered “normal” for human beings. According to the research of Ivayla I. Geneva et al. [43], which presented a systematic review of evidence-based normal temperature ranges, temperature is a nonlinear function of several variables, including age, gender, ambient temperature, health status, the person’s circadian rhythm, among many others. The most important variables are the site of measurement and the patient’s age [43]. Many studies have developed and reported in the literature wearable systems that provide both skin and core temperature measurements in real-time [44,45,46,47]. 

Boano et al. [45] demonstrated an unobtrusive and body temperature monitoring system based on a body sensor network with sensor units attached to the skin. The authors intended to study the effects of sleep deprivation in the human circadian rhythm. Sleepiness has proved to induce significant changes in core body temperature. To measure the accuracy of the designed prototype, preliminary experiments were made to monitor circadian rhythms of a subject for 24 hours, as well as the corresponding mental activity. In the first experiment, the sensing unit was placed in the non-dominant hand, and in the second, skin temperature was measured at the temple of the subject. These units could measure and send the temperature information to another body-worn central unit with more processing power. A computer was used to receive the data from the central unit and sequentially communicate with a healthcare facility over the internet. The data was compared with a thermometer and an accuracy of 0.02 °C over the temperature range of 16–42 °C was achieved by the authors. 

Chika Sugimoto and Ryuji Kohno [48] developed a wireless sensing system to monitor an individual’s thermal physiological state by using an ear-worn temperature sensor, two thermo-hygrometers and four skin temperature sensors. The sensors transmitted data wirelessly in synchronization with each other to a computer for further analysis of tympanic temperature, skin temperature, humidity and temperature.

Regarding the acquisition of core temperature, which is an indicator of thermal stress, thermal comfort level and health status, Looney et al. [49] presented an algorithm based on an extended Kalman filter that estimated the body core temperature from the heart rate. According to the authors, the algorithm could estimate human core body temperature in real-time with a high level of accuracy.

A selection of the available techniques for vital signs monitoring reported in the literature is presented in Table 2. 

## 3. Body Motion and Daily Activities Monitoring

In addition to the monitoring of physiological signs, there is another equally important concept in AAL, which is the monitoring of gait parameters that characterize a person’s locomotion, and the monitoring of activities of daily living (ADL). 

Monitoring individual’s walking patterns can provide important data about their health conditions. Gait disorders, for example, may be caused by neurological conditions, orthopedic problems and medical conditions [50]. Vision-based systems and cameras are very useful to monitor gait activities and detect physical impairment. However, despite the accuracy achieved with these systems, there are some constraints that limit their use. Some limitations include privacy issues, and the fact that the user must always remain in line of sight and within a specific range from the camera, which somehow prevents continuous monitoring of gait activity. Alternatively, wearable motion sensors based on accelerometers and gyroscopes are a great solution to assess gait dynamics. Several key features can be extracted from the sensors based on the linear and angular motion measurements obtained from body kinematics [9].

Following this method, Bertolotti et al. [51] developed a versatile wearable device based on an inertial measuring unit (IMU) that extracts information from trunk and limb movements. This allowed the motor and balance control ability assessment, based on linear accelerations, angular velocities and heading. The authors compared the centre of mass (CoM) displacements on the horizontal plane (X and Y) with that obtained using the centre of pressure (CoP) of the Wii balance board (WBB), which has been considered a reliable tool for CoP measurements. Another project based on IMUs and a gait recognition algorithm was presented by Thanh Ngo et al. [52]. Three inertial sensors were placed at three different positions and orientations on a person’s waist, and the proposed algorithms were able to identify gait action classes, such as walking on a flat ground, go up/downstairs and go up/down a slope. Carnevale et al. [53] also referred the wide use of wearable systems based on IMUs in the literature, namely for shoulder kinematics and musculoskeletal injuries assessment. The authors also reported different configurations found in several studies regarding the number of sensors and their placement on the human body.

Monitoring systems for assisted homes may include the capability of recognizing behaviors and certain patterns of human daily activities, in order to mediate and detect possible symptoms of a certain disease, whether mental or physical. ADL address the daily life activities of people in their own home environments, without requiring any assistance to execute them. The ability to perform such elementary routines while aging determines the person’s physical and psychological health status and their ability to live independently. Such monitoring helps to track possible developments of mental illnesses associated with aging, namely Alzheimer’s, Parkinson and other levels of dementia. ADLs mainly comprise activities that are based on hygiene, mobility levels, dressing, eating and continence. In short, ADL addresses any task associated with physical self-maintenance that is essential to ensure the health and well-being of an individual.

The most used technologies for indoor localization are included in Table 3, and are expressed by mechanical, magnetic, acoustic, radiofrequency and light-based methods.

There are many factors to consider when monitoring such activities, namely the choice of technology to be used for activity recognition, as well as its ability to be deployed in households, ease of use of the implemented system and their privacy levels. Several studies regarding the monitoring of the user’s behavior and daily routine are expressed by systems based on wearable sensors, video surveillance, appliance monitoring and distributed sensors throughout the house [57]. However, the implementation of such sensing technologies may raise several privacy concerns, due to their ability to assess relevant information about people’s lives. In fact, the most accurate mechanisms for activities recognition and monitoring include video-based strategies, such as video-cameras or thermal-cameras. The implementation of such technology is not always accepted by the users, and most rooms cannot be accessed due to heavy privacy violation. As an alternative, the use of low-informative sensors, such as magnetic switches, infrared motion sensors, pressure sensors, ultrasonic sensors, among others, is a better strategy that preserves the desired privacy levels. Despite being less informative about human activities, the installation of multiple instances of these sensors throughout the house and the implementation of sensor fusion can overcome that limitation.

Debraj et al. [66] developed a system for recognizing a person’s in-home activities and body motion (via an accelerometer and gyroscope). The location of the user is given by Bluetooth beacon location tags, placed in each room. A context-based activity classifier was designed based on this multimodal sensors and body wearables, and an accuracy of more than 80% on classifying 19 in-home activities was reached with the proposed system.

Chernbumroong et al. [67] developed a multisensor system to be worn on both wrists for activity recognition. Accelerometer and gyroscope sensors related with movement were worn on the dominant wrist, as well as a light sensor and a barometer. Temperature and altimeter sensors were worn on the non-dominant wrist. The sensor’s location did not interfere nor interrupted the user’s daily activities. While the accelerometer was considered the most effective sensor for detecting and recognizing activities, the inclusion of all other additional sensors further improved the accuracy of activity classification. 

Another system, combining acceleration data with vital signs to achieve a more accurate recognition of performed activities, was proposed by Centinela [68]. The system did not classify specific ADL, but recognized physical activities such as sitting, walking, running, ascending and descending. Data acquisition devices were based on a smart phone and a single sensing device. As concluded by the authors, the acquisition of vital signs was also useful to discriminate between the different performed activities.

The previously mentioned systems are mechanical-based and use IMUs, such as accelerometers and gyroscopes, to determine position and angular motion of a target relatively to its previous position. However, these methods are obtrusive since they need to be attached to the surface of a target, in this case the human body. Nonwearable sensors are less intrusive and can be placed in stationary locations of a house or a room. They can provide significant information about performed activities whether by monitoring the operational status of objects, detect movement in a room, measure room temperature, monitor the opening/closing of doors and so on.

Fleury et al. [62] developed a system for detecting ADL based on different sensing technologies. Infrared presence sensors were used for location purposes (e.g., detection of movement) and placed at strategic locations; door contacts were fixed on relevant home appliances (e.g., fridge, cupboard and dresser) to detect its usage and to calculate the percentage of time in which they are open or closed; microphones were used to process and identify different sounds of daily living activities (e.g., speech, door shutting, phone ringing, walking sound, among others); and wide-angle web cameras were deployed to timestamp ADLs for supervised machine learning algorithms. The authors also placed temperature and hygrometry sensors in the bathroom to detect activities related to hygiene. Additionally, a wearable kinematic sensor with accelerometers and magnetometers was also implemented to detect and classify transitions in posture and walking periods. 

The authors in [69] recorded data relative to ADL in two different households during one week. The user’s localization was based on multiple low-informative sensors fitted in the house, such as magnetic contact and motion sensors, microphones and power meters. These sensing technologies were considered enough to get an insight of the user’s activities and were strategically placed over different rooms of the house.

The Washington State University’s project named CASAS (Centre of Advanced Studies in Adaptive Systems) [70] was meant to develop a smart home and detect broad activities such as eating, sleeping or wandering. The smart apartment was populated with various types of sensors to detect movements (mainly by infrared/light sensors), the usage of certain home appliances and items, energy consumption and environmental temperature and to perceive the state of doors and lights. This project implemented machine learning techniques for human activity recognition, based on generated events from the sensors. 

H. Ghayvat et al. [71] implemented a lifestyle monitoring system to detect ambiguities and anomalies in the activity of daily living (ADL) patterns that may indicate possible symptoms of disease. Several sensing units were distributed through the AAL environment and sensor data fusion techniques were implemented to enhance the system’s efficiency. Information regarding the daily activities and behavior was collected from four elderly houses during three hundred days. 

Considering the implementation of sensor networks for detection of behavioral patterns, the use of light dependent technologies, such as infrared and photoelectric sensors, can lead to some issues. These sensors may produce wrong outputs (e.g., false positive or false negative triggers). Failure in these sensors can lead to a misinterpretation of the subject’s health status and bring negative consequences to their health. Regarding this limitation, Nancy ElHady et al. [72] made a systematic literature review on sensor fault detection and fault tolerance in AAL environments. A sensor failure in a AAL environment can be considered as a fault if the sensor has stopped responding (fail-stop failure) or if the sensors are still responding but the reported values are not representative of the measured variable, nor the type of event that is supposed to be detected (non-fail-stop failure) [73]. The last type of failure can be caused by external factors that trigger these false events, such as changing the location of the furniture where the sensors are installed to a different area, or slightly changing the position of sensors, or due to the covering of sensors whether by unwillingly placing objects in front of them or by other environmental interferences [73]. The authors concluded that this research area still needs an intensive investigation in order to ensure the implementation of robust sensor fault detection systems in AAL environments in the future.

Regarding radio communication protocols, several have been used to provide indoor localization services, such as Bluetooth (IEEE 802.15.1), Radio Frequency Identification Devices (RFID), Ultra Wideband (UWB), Wi-Fi (IEEE 802.11) and ZigBee (IEEE 802.15.4).

Bluetooth, or IEEE 802.15.1, is a strong candidate for indoor localization systems and it is used in a large number of studies [66,74,75,76,77,78]. Bluetooth is a standard based on a wireless radio system and it is designed for short-range wireless communications. It is mainly oriented to establish wireless connections between closely connected devices and is widely used in IoT systems due to its high energy efficiency. Bluetooth Low Energy (BLE) provides improved speed, greater coverage range and versatility when compared with its older version, Bluetooth Classic. This protocol is best used for localization purposes when beacon communication is used. Devices and sensors that use BLE interface can be placed in different areas and programmed to send broadcast messages, to be received by listener devices (e.g., a mobile device or sensor node used by the patient) [79]. It is then possible to know the approximate location of the user based on the received signal strength indicator (RSSI), which is used to estimate the distance between the transmitter and receiver device. This technology has been widely used in the marketing industry for costumer engagement and proximity marketing at stores, museums and events. Commercially available BLE based protocols include iBeacons (by Apple Inc.) and Eddystone (by Google Inc.), which are specially designed for proximity detection.

Solutions based on Bluetooth beacon technology for indoor positioning estimation were addressed by Xin-Yu Lin et al. [80], which implemented a mobile-based indoor positioning system based on the iBeacon solution. The goal of this research was to help medical staff track the locations of their patients inside a hospital. To evaluate this approach, the beacons, with transmitting signals ranging about 30 meters, were placed at the ceiling of four hallways and two rooms of an experimental test-bed environment. A mobile application was used by the patient to collect the signals from the beacons, based on RSSI values. The authors claim to achieve an accuracy of 97.22% on classifying the location of the patient.

The previously mentioned project from Debraj et al. [66] also used Bluetooth beacon technology for ADL recognition. The beacons were placed on each room (e.g., bedroom, kitchen and bathroom) of an inhabited home and served mainly as an indicator of the user’s presence in a room. The receiver device consisted of a smartphone using an RSSI-based algorithm for estimating location context, based on the closest proximity of the patient’s smartphone to a Bluetooth’s beacon.

This technology does not provide an accurate and precise location of the user and it is mostly used for context aware proximity services, which is satisfactory for AAL environments. 

The use of RFID is also a great solution to monitor in-house daily activities that require proximity to certain appliances and furniture, as presented in the literature [81,82,83,84]. This protocol is based on electronic tags (RFID tags) that exchange data through radio waves to RFID readers. These tags are made up of an antenna and an integrated circuit. The first component allows the transmission and reception of radio frequency (RF) waves, while the second one is used for processing and storing data, as well as for modulating and demodulating radio waves. Considering the detection range and power source, there are three types of RFID systems: active, passive and semipassive [85]. Active RFID tags need an internal battery source and can operate at a range of hundreds of meters from the RFID reader. They work in the ultra high frequency (UHF) and microwave frequency range and are mostly used for localization and tracking of objects [86]. Passive RFID tags have no internal energy source (current is induced on the antenna by the RFID reader) and have a limited range between 10 cm to a couple of meters. They can operate in the low, high, UHF and microwave frequency range and, despite not being good for indoor localization systems, due to its limited range, they can be used to monitor the usage of certain appliances at home. Semipassive RFID tags are like active tags because they have their own energy source, which is not used when communicating with the reader, like with semipassive tags. The battery is only used to power up the microchip, which helps to increase the amount of energy reflected from the RFID reader to the RFID tag, thus allowing a higher read range than normal passive RFID tags.

There are two ways in which RFID systems can be used for indoor location [83]: the RFID tag acting as a target and carried by the patient is sensed by RFID readers distributed in specific areas of the house, or, the RFID reader is attached to the patient and senses different RFID tags that are placed in specific places of the house. A more practical case regarding the use of RFID technology applied to AAL environments is the project HABITAT (Home Assistance Based on the Internet of Things for the Autonomy of Everybody) [87], whose main objective was to monitor and assist elderly in their daily life activities. The developed system was based on a RFID system for indoor localization. Multiple active tags were worn by the patient and two or more RFID readers were strategically placed on the walls. The system showed a good estimation of the user’s location, presenting an average error of about 18 cm.

Ultra Wideband (UWB) is another radio technology that is widely used in indoor localization applications [88,89,90]. It has been used for short-range communication systems and it is based on the transmission of short pulses across the wide spectrum frequency with a time period of less than 1 nanosecond (ns) and over a high bandwidth (500 MHz) [86]. Its different signal type and radio spectrum makes UWB immune to interference from other signals, which help this technology achieve its precision and accuracy in indoor localization systems. However, the UWB dedicated infrastructure and location tags are expensive. Other drawbacks include the need of having at least three receivers to receive the signals from the tags, a higher level of complexity on its installation, since UWB reader locations need to be carefully chosen for the system to work, and its susceptibility to interference under the presence of metallic materials [91]. The authors referred in [48] used an UWB positioning system to track the user’s location without hindering their mobility. While studying the relation between the performed activity and location, the authors could monitor four different activities considered useful to help prevent heat strokes on elderly (e.g., fluid intake, having a meal, fluid elimination and going outdoors).

G. Oguntala et al. [92] conducted a survey on the most appropriate indoor technologies for reliable real-time positioning and classified eight radiofrequency based indoor technologies (magnetic-based, ultrasound, infrared, Bluetooth, Wi-Fi, ZigBee, UWB and RFID) on eight different performance metrics: scalability, accuracy, complexity, robustness, reliability, energy efficiency, cost and throughput. RFID technology had the best overall classification, presenting a better performance in terms of cost and energy efficiency. On the other hand, infrared presented the lowest score, and was classified as the less accurate method. 

Data fusion between both radio frequency and mechanical-based systems can greatly improve the overall classification of daily activities, as verified by Y.-J. Hong et al. [93]. The authors used RFID and accelerometers to detect and recognize the user’s activities on their daily living environment. The accelerometers were located on the wrist, tight and waist. An RFID reader was also placed on the wrist, while RFID tags were located on daily used objects. Accelerometers were also added in order to classify five different states of the human body, such as standing, lying, walking, sitting and running. The correlation between a body state and the use of an RFID-tagged object increased the system’s efficiency on recognizing user activities based on the handling of daily-used objects. Data fusion of both accelerometer and RFID measurements provided better accuracy on detecting specific activities (e.g., drinking while standing; reading while sitting; brush hair while standing, etc.), reaching an overall recognition rate of 95% of eighteen ADL.

## 4. Indoor Environmental Quality Monitoring

Monitoring of human physiological status is the most important factor to consider when creating an AAL system, as it helps diagnosing human health conditions and prevent possible at-risk situations. However, environmental conditions also play a vital role on the population health and well-being and can be remotely monitored in real-time to prevent dangerous and adverse situations, namely associated with poor air quality. Indoor environmental quality (IEQ) is an indicator of the general quality conditions of indoor environments that may have an impact on human’s health. The IEQ indicator is composed of multiple subdomains [94], including air quality, lighting quality, noise levels, thermal comfort, among others. This section aims to address the most important IEQ factors and how their monitoring and control can be achieved.

### 4.1. Indoor Air Quality

Air pollution is one of the greatest risks for human health. It can potentially cause numerous respiratory problems such as asthma, chronic obstructive pulmonary disease (COPD), allergies and, in a more extreme case, lung cancer. While most people are aware that outdoor air pollution has a major impact on their health, few have the idea that indoor pollution can be far more harmful. According to the United States Environmental Protection Agency (EPA) [95], indoor pollution levels can be 2–5 times higher than at outdoor environments. IAQ monitoring systems are essential in every smart home and AAL environment since the population usually spends approximately 90% of their time inside buildings.

Particulate matter (PM), ozone (O_3_), sulphur dioxide (SO_2_), nitrogen oxides (NOx) and carbon monoxide (CO) are the most common air pollutants present in urban areas and can either be formed by both outdoor and indoor sources of pollution [96]. According to [97], the air contaminants that are most linked to asthma-related hospital emergencies comprise PM_10_, NO_2_ and O_3_. Additionally, outdoor air pollutants greatly affect indoor environments, since the air exchange between these two environments is constantly done through mechanical ventilation and natural ventilation [98]. However, most pollutants created by indoor sources have a greater impact on indoor air conditions. These pollutants usually come from combustion sources, cleaning products, air conditioners without proper maintenance, smoke, cooking oils, building materials and many other indoor sources. The acceptable limits of concentration for some of these IAQ contaminants is presented in Table 4.

Apart from air pollutants, other factors, such as indoor temperature and relative humidity, need to be considered regarding asthma distress prevention and well-being. A temperature between 15 and 30 °C and a relative humidity between 40% and 60% is considered the ideal for indoor environments [100], as it minimizes most adverse health effects. Values of relative humidity above 60% will turn the air harder to breathe—besides narrowing and tightening the airways, humidity also makes the air stagnant and traps pollutants and allergens, which can help trigger asthma attacks [101].

Different IAQ monitoring systems have been proposed in the literature, along with different distributed sensing solutions. Considering the adoption of primary-prevention strategies to help avoiding the triggering of potential asthma attacks and COPD, the authors in [102] developed a distributed smart sensing network for IAQ assessment. Gas sensing units based on semiconductor heated sensors and electrochemical cells were used to measure gas concentration, and an additional channel was implemented to measure temperature and relative humidity. The system could estimate the air quality index of the indoor environment based on the measured gas concentration. A smartphone application was developed to notify the user for possible asthma and COPD attacks, based on previously stored threshold values. However, strategies to improve indoor air conditions rely on user actions (e.g., manually opening the window to allow air flow and displacement of indoor pollutants), which can be a limitation for patients with low mobility.

Automatic adjustment of IAQ based on the use of actuators (e.g., air conditioner and mechanical ventilation units) is one of the great benefits of home automation systems. Following this strategy, Salamone et al. [103] implemented a smart object that helped improve the overall air quality by automatically controlling the air exchange system. The air quality evaluation was solely based on the measurement of concentrations of CO_2_. To evaluate whether the applied ventilation strategy was reliable, and to establish CO_2_ concentration limits, the authors considered the calculation of the difference in CO_2_ concentration between indoor and outdoor air. The implemented system was tested in real working conditions and it was considered suitable in optimizing IAQ.

Considering the monitoring of a wider range of air pollutants, Nikolas Vidakis et al. [104] developed an embedded monitoring system to measure air quality parameters such as temperature, humidity, as well as CO and ozone. The system was designed to be deployed at spaces with limited human intervention, where remote control and monitoring is required. Therefore, the authors included measurement capabilities of each node’s current consumption in real-time, as well as the development of a web application for continuous monitoring, data visualization and node management. The system also came with a notification alert mechanism for when measured values of IAQ were considered unsafe.

Based on a WSN, Xinrong Li and Sherin Abraham [105] proposed an air quality assessment system that could simultaneously obtain CO_2_, CO, ozone and volatile organic compounds (VOC), as well as temperature and relative humidity, from different locations. The calibration of the gas sensing units was achieved by comparing the acquired data from the sensors with a professional air quality measurement system, capable of detecting the same types of gas.

Jung Kim et al. [106] developed a gas concentration monitoring system for a wider range of air pollutants—ozone, CO, NO_2_, SO_2_, VOC and CO_2,_ and particulate matter (PM)—in a real-time basis, providing overall air quality alerts. These authors took into consideration of several aspects, such has the optimal number of required sensor nodes and their correct placement in the environment, according to the type of pollution sources. The wide range detection of air pollutants made the system suitable for both indoor and outdoor environments. Their sensor node computing platform allows scalability of the system and its integration in IoT applications.

The authors in [107] presented an IoT solution focused on monitoring air quality parameters that directly affect people with asthma and COPD. The system provided different warning notifications through visual alert mechanisms based on smart lighting, as well as notifications on the smartphone, when air conditions were considered harmful. The IAQ analysis was performed by the deployment of a WSN composed of a particle counter to measure PM concentration levels, three semiconductor heated gas sensors with relative sensitivity to the air contaminants presented in Table 4, and a temperature and relative humidity sensor. An actuator node based on a ventilation unit was also deployed to help changing and improving IAQ levels automatically whenever the imposed thresholds for temperature, relative humidity or gas concentration were exceeded. A web-based application was developed by the authors to allow real-time monitoring of each measured IAQ parameter and remote control over the system.

### 4.2. Indoor Lighting Quality and the Impact of Noise in Health

Poor air conditions do not only affect individuals with respiratory illnesses. Common symptoms that are often linked to poor air quality for most people include headaches, fatigue, shortness of breath, coughing and dizziness [95]. However, these are not necessarily caused by poor air quality. ILQ and indoor noise levels also have a great impact on human health, and thus, may be the cause of the manifestation of such symptoms. ILQ plays an important role in an individual’s visual ability and has several positive biological effects. The benefits of adapting both light levels and color temperature throughout the day in indoor environments are numerous. The recommended light levels [108] for each area of the building and for each type of working activity must be considered. Adequate lighting levels during the day and night can regulate circadian sleep–wake rhythms and vastly improve an individual’s health, productivity and comfort. Circadian lighting is a concept that is becoming often present in various sectors, from healthcare to corporate [109]. It follows the circadian rhythm, a 24-hour internal clock that cycles between sleepiness and alertness at regular intervals. Lightness and darkness have a direct impact on this sleep–wake rhythm. The eyes send signals to an area of the brain called hypothalamus, which will report if it is night-time or daytime. The hypothalamus, in turn, controls the amount of melatonin that needs to be released, associating sleepiness with darkness and alertness with lightness [110]. Given that most of the population does not have access to natural light in their working environments and at home, they are often exposed to non-natural electric light. Electric light is usually kept mostly within certain wavelengths of blue light, which can lead to negative impacts on melatonin production. Smart lighting systems have been recently helping to address these problems [107] and can be provided by some commercial products such as Yeelight LED Smart Bulb [111] and Philips Hue [112]. Capable of changing its light temperature color and intensity, these systems can be used to support human health and regulate sleep–wake rhythms.

Another important factor that has a remarkable impact on human health is the daily exposure to high levels of noise. With population growth, increased vehicular traffic and industrial activities, noise is increasingly present in the daily lives of millions of people. Although the notion of noise may vary from individual to individual, depending on their subjectivity or auditory sensitivity, prolonged exposure to sounds above 80 dB may cause permanent damage to the auditory system. Guideline values of noise for specific environments, such as recommended by the World Health Organization [113], must be followed in order to minimize the underlying critical effects on human health. Problems such as sleep disturbances, stress, difficulty in communication between people and loss of concentration are among the most frequent effects caused by this physical agent [114,115]. Therefore, monitoring noise levels and notifying the individual for when values exceed an acceptable threshold for a certain period of time is an equally important factor in preventing hearing damage and helps to ensure productivity, well-being and human health.

## 5. The Role of Artificial Intelligence (AI) on Smart Tailored Environments

Activity recognition, specially ADL, and behavior pattern classification is at the core of every AAL system, since it provides information about cognitive health progression or degradation. Such a health assessment is critical for the doctors or family to decide whether the patient should move to an assisted living environment with constant supervision or to other care facilities. As previously mentioned, monitoring of the user activity and behavior is obtained through indoor localization technologies that can be expressed by several sensors distributed through the house, or by other wireless systems based on radio frequency communication. Information from sensors, which is often considered high-level, cannot be, evidently, obtained by a direct observation of their raw data. It must be processed by suitable algorithms usually based on machine learning, signal processing and data analysis [3].

The application of such algorithms depends on the chosen activity recognition approach. Visual based indoor localization, such as camera/video recording, requires computer vision techniques to recognize activities from several visual observations on the user’s actions, gait patterns, as well as environmental changes [116]. With the usage of sensor network technologies, for instance, data must be analyzed through data mining and machine learning algorithms applied to build activity models that later will be used as the basis of ADL recognition.

There are two categories in machine learning algorithms used for activity recognition, where the differentiation between the two lies on how the user’s activities and their ADL profile are represented and modeled. The first category refers to the generative approach, which consists on creating a statistical model of the joint probability distribution of samples and activity labels. The most typical generative models include the hidden Markov models (HMM) and Bayesian networks. The second approach is a more heuristic approach and is based on creating a model of the conditional probability of the activity labels, given the samples [117,118].

Discriminative models include support vector machines (SVM), which present high accuracy and good performance when a limited dataset is considered, conditional random fields (CRF), k-nearest neighbor algorithms and artificial neural networks (ANN), with the most prominent ones being recurrent neural networks (RNNs). Several datasets of smart home projects are publicly available and can be used for testing the most suitable machine learning algorithms for ADL. In most of this datasets, human activities are perceived by a sequence of state-changes expressed by the activation of several sensors (e.g., infrared motion sensors, pressure sensors and so on) installed on everyday used objects. Some datasets include CASAS dataset [119], MavHome [120], ARAS [121], MIT Activity Dataset (Tapia) [122] and Kasteren [123], among others.

The CASAS project [70] created activity recognition software that provides real-time activity labeling of sensor events (e.g., cooking, eat, enter home, sleep, work, etc.), based on a support vector machine (SVM). The analyzed data was based on the sequence of sensor events (e.g., “ON” and “OFF”) of several motion sensors distributed throughout the house. Data was collected from 18 separate smart apartments, having a single resident each. The weighted average accuracy on classifying activities was 84.0%, considering the implementation of the system in different home settings. The authors also tested other machine learning algorithms, including hidden Markov models and naïve Bayes classifiers. However, the SVM achieved better performance on classifying such activities in real-time than the other approaches.

Fleury et al. [62] made ADL classification based on SVM as well. Bayesian classification and neural network methods were not suitable given the small number of collected samples. The authors in [48] used a hidden Markov model (HMM) to recognize behavioral patterns expressed by state sequences. Activity recognition was based on the user’s location, which was obtained by an impulse radio UWB positioning system.

Yegang Du et al. [124] developed a three-stage framework for recognition of human activities, able to predict the next probable activity. This recognition was based on the manipulation of daily used objects (e.g., chair, bed, sofa, toothbrush, knife, etc.). The detection of its usage was done by attaching passive RFID tags on the objects. Information regarding the handling of one object was given by transforming the physical-signal data to binary-state data, considering that the physical manipulation of one object is perceived by the analysis of the radio frequency phase value of RFID tags [125]. For human activity prediction, time sequences were considered, as certain activities tend to happen right next to a previous activity (e.g., watching TV after having dinner). Long short-term memory (LSTM), a subset of recurrent neural networks (RNN), was used for activity prediction, as well as for object-usage. The authors achieved a recognition precision of 85.0% and prediction accuracy of 78.3%. Their solution showed a stronger performance and accuracy than the classical naïve Bayes method [124]. 

The authors in [69] evaluated different methods of classifying ADL. The classified activities included going to the toilet, taking a shower, going to bed, eating, drinking, etc. SVM, random forest, HMM and Fisher kernel learning (FKL) classifiers were tested on three data sets with different types of sensors at each different location. The first dataset was from Kasteren [123]. It described the daily activities performed by a single person in his apartment. All the used sensors (including motion, pressure and reed switches) gave binary outputs. Around 2120 sensor events were generated in his apartment during 25 days [126]. The second dataset was from the CASAS project [119] and the third one was collected by the authors in two different households during a week. Those households generated around 7856 sensor events, for the first, and 8618 sensor events for the second. As concluded by the authors, the hybrid generative and discriminative method, FKL, presented a better performance for the three datasets, when compared with HMM, SVM and random forest algorithms.

Considering ADL recognition systems based on motion, with data extracted by accelerometers and other mechanical-based sensors, Chernbumroong et al. [127] were able to detect nine different ADL of an elderly person based on the information provided by wrist-worn multisensors from a sports watch, such as a temperature sensor, accelerometer and altimeter. When compared with neural networks, SVM proved to be the best algorithm for the classification of activities, with an overall accuracy of 90.23%. Future work [67] included the addition of four more sensors— the heart rate monitor, light sensor, gyroscope and barometer—to improve the activity classification accuracy. By using the SVM classification model, which was still considered the best classification algorithm for their dataset, the authors achieved approximately 97.20% accuracy when classifying activities.

Davis et al. [128] evaluated three machine learning algorithms—SVM, HMM and ANN—on a dataset based on information collected with an accelerometer and gyroscope of a smartphone. SVM and ANN classifiers achieved a good performance (97.6% and 91.4%, respectively), but the combination of both SVM and HMM methods vastly improved detection accuracies to 99.7%.

Other classifiers, such as decision tree algorithms and its variants, have also been used for human activity recognition systems [129,130,131].

Table 5 summarizes machine learning classifiers that have been used in the literature for ADL recognition.

Besides having a strong presence in ADL recognition, machine learning techniques are also applied to extract relevant information about the physiological status provided by wearable biomedical sensors. In addition to HRV analysis, mental or emotional strain can be estimated by applying machine learning methods to physiological data. Emotion estimation, for example, can be achieved by analyzing physiological features obtained from ECG, RR, galvanic skin response (GSR) and skin temperature, as proposed by Kukolja et al. [132], or by analyzing PPG and GSR signals together, as proposed by Domínguez-Jiménez et al. [133]. A comparison of the best machine learning techniques to detect psychophysiological stress was studied by Smets et al. [134], based on physiological responses obtained from ECG, GSR, RR and skin temperature in a controlled environment. Previous studies [30,135,136,137] have also considered machine learning to classify PPG and ECG signals and detect cardiac arrhythmia types, which provides critical information to detect heart diseases and help medical entities to find the best treatment for the patients. Among many other applications of machine learning technology on physiological data, blood glucose estimation [138] and hemoglobin level prediction [139,140], for example, are also presented in the literature.

## 6. The Importance of Exergames and Immersive Environments for Physical and Cognitive Stimulation

With the increase of life expectancy and retirement age over these recent years, the risk of mental illnesses, particularly dementia and strokes, has been a raising risk for most of the elderly population. Not only do the risks of mental illness arise at this age, but there is also an emergence of negative events, such as the loss of a loved one, lack of close family ties, loneliness, social isolation and decline of mobility and physical exercise. These issues lead to an urgent need of providing healthcare systems that can contribute to medical rehabilitation and enhance social well-being among the elderly. The exergames, which combine physical exercise with digital gaming, have proved to bring great benefits to the participants’ physical, cognitive and psychological well-being [141,142]. Most importantly, the elderly can use these systems in their own home, where they feel emotionally more comfortable and where the rehabilitation process can be more efficient. Many studies have proved the great benefits of using exergames in improving participants’ physical and psychological health.

Jinhui Li et al. [141] conducted a literature research on exergame studies and concluded that the interaction of elderly population with these type of video games have promising results regarding the enhancement of social well-being, including the increase of positive attitudes and social connection, and also reduction of loneliness.

Chan et al. [143] studied the influence of virtual reality (VR) technologies in cognitive functions of older adults and concluded that VR based training programs significantly improved repetition and memory retention compared to usual programs. With a special focus on providing a cost-effective way to support mental wellbeing and physical and mental rehabilitation for elderly at home, E. Vogiatzaki and A. Krukowski [144] proposed an automated home system that combines augmented reality and virtual reality gaming, multimodal user interfaces and innovative embedded microsensor devices combined with a personal health report system (PHR). This system was intended to support the delivery of individual and patient-centered electronic health services at home, hospitals and other types of environment, and its usability was confirmed by technical validation tests.

Considering the design of tailored VR games for people with dementia, Hodge et al. [145] proposed some directions when designing a VR game for people with dementia, based on two workshops that involved VR experiences carried out in a local dementia care charity. Directions include paying attention to the physical design of the headset, so it is not too heavy and uncomfortable for the user; to include shared experiences, such as screen displaying the same view as the headset, so keepers and loved ones can also interact and guide the person with dementia; to use the full spectrum of sensory experiences, such as including ambient sounds and music in the game, to make the best use of senses and abilities that are still remaining in the person with dementia; personalize the game to each individual, according to his own preferences or allow the keeper to configure the environment; and finally to position the person with dementia as an active participant.

The creation of immersive environments has been addressed by several AAL projects, some of which have been supported by the European Active Assisted Living (AAL) Program [146]. SENSE-GARDEN [147,148] is a project based on the development of immersive environments that provides different stimuli for basic senses, such as balance, smell, touch, hearing and sight. These environments integrate music, films, pictures and scents, and are specifically tailored for the individual, as they automatically adapt to their personal memories and preferences. All this was achieved by the design of a virtual space, composed by: a reality wall with projection of landscape videos with familiar scenarios; an augmented reality game to improve balance and physical activity stimulation; an interactive touchscreen showing family photographs; a stationary bicycle placed in front of a film; sound speakers playing background soundscapes and familiar music and a dispensary system releasing familiar scents [147].

As a tool to assist people with cognitive disabilities, Covaci et al. [149] proposed a VR system to provide support for planning tasks and for context-based learning. Although the project was aimed for people with Down’s syndrome, it could be inserted into any AAL environment for other potential users. An example was the development of a VR application to train the users to navigate in the outdoor environment. Routes were realistically built using Google Street View API and the virtual trip could be customized by caregivers and family by adding personal information, such as photos or images, or mark important places and locations along the route. The creation of exercise environments with manipulated auditory and visual feedbacks permits personalized training, which allows this immersive environment to be adapted to any individual.

## 7. Conclusions

As technology advances, there are always innovative solutions for assisted living systems and healthcare assessment. The proposal of new architectures is essential to reach healthy, elderly or disabled individuals and improve their quality of life. AAL systems are based on the architecture of an IoT-based healthcare system and have a special focus on providing personalized and assistive services for their inhabitants. Through the monitoring of several physiological parameters and behavior patterns, these environments have the main purpose of determining a person’s physical and mental health status and help to extend the period in which a person can live independently, in their preferred environment.

Physiological data can be acquired by smart biomedical sensors, either wearable or non-wearable devices. Such acquisition systems can determine the most relevant human vital signs, including body temperature, pulse, respiratory rate and blood oxygen saturation, which can be remotely monitored by caregivers and healthcare entities. Information about the mental state of the user can be given by the monitoring of their behavior, where changes in daily activities that deviate from a behavioral pattern can indicate a possible disease. This is achieved by the integration of indoor localization systems that can provide information about the user’s location, along with the most suitable machine learning and signal processing algorithms for activity recognition and classification.

Different solutions regarding the development of healthcare systems and assistive environments are proposed in the literature. Most projects are effective in monitoring human physiological status with the use of body sensor networks and others to monitor the activities of daily living using indoor localization techniques and machine learning algorithms. However, few or non-existent, consider the creation of smart assistive environments that put together body sensor networks for vital signs monitoring, smart sensors and environmental sensing for indoor environmental conditions assessment, activity recognition and artificial intelligence software modules, and VR/AR implementations for cognitive and physical stimulation as part of a fully integrated AAL system.

This paper addresses all the relevant components and services used for the implementation of a healthcare assessment system that will best serve the needs of an elderly population. A review of the current AAL solutions presented in the literature was carried out, including an analysis of vital signs monitoring systems, suitable indoor localization technologies, daily activities and gait monitoring systems, as well as the most used machine learning classifiers for activity recognition. The importance of monitoring indoor environmental quality and how it can be improved with the implementation of actuator nodes and prevention strategies was also addressed. Due to the raising risk of the development of mental illnesses in the elderly population, several solutions concerning physical and cognitive stimulation were analyzed as an additional and essential component that should be included in an AAL environment.

## Figures and Tables

**Table 1:** Heart rate variability parameters.

Parameters	Units	Definitions
Time-domain analysis		
Mean HR	bpm	Mean of heart rate values
Mean RR	ms	Mean of RR interval time series
SDNN	ms	Standard deviation of successive NN intervals
RMSSD	ms	Root mean square of successive NN interval differences
SDSD	ms	Standard deviation of differences between adjacent NN intervals
NN50	ms	Number of successive intervals differing more than 50 ms
Frequency-domain analysis		
VLF, LF, HF	ms^2^	Power in very-low, low, and high frequency range, respectively
LF/HF	-	Ratio between LF (ms^2^) and HF (ms^2^)
Non-linear methods		
ApEn	-	Quantifies the regularity and complexity of the time series. It measures the unpredictability of the variation of successive RR intervals.
SampEn	-	Improved evaluation of time series regularities (modification of ApEn).
DFA	-	Quantifies the presence or absence of fractal correlation properties of time series data. It permits the estimation of long-range correlation in non-stationary time series [42].

**Table 2:** Vital signs monitoring techniques.

Method	Definition	Monitored Signs	Reviewed Works
Electrocardiography (ECG)	Measurement of electrical activity of the heart	HR, RR	[15,17,18,19,20,21,37,38,40]	
Photoplethysmography (PPG)	Optical measurement of blood volume changes in microvascular bed	HR, SPO_2_, RR, Blood pressure	[27,28,29]	
Seismocardiography (SCG)	Measurement of microvibrations of the chest wall produced by the heart contraction and blood flow	HR, RR	[22,40]	
Ballistocardiography (BCG)	Measurement of hole-body microvibrations associated with the cardiac cycle	HR, RR, Blood pressure	[31,32,33,34,35,36]	
Contact thermometry	Temperature measurement based on conductive heat changes between the surface of skin and a temperature sensor	Skin temperature	[44,45,46,47,48]	

**Table 3:** Classification of indoor tracking and localization technologies.

Mechanical	Magnetic	Acoustic	Radio Frequency	Light
Pressure sensor Proximity sensor Vibration sensor Accelerometer ^1^ Gyroscope ^1^	Magnetic field sensor [54,55,56,57]	Ultrasonic sensor [58,59,60,61] Microphone [62,63]	Wi-Fi Bluetooth ZigBee [64] RFID ^1^ UWB	Infrared sensor Photoelectric sensor Camera/Video recording [65] LIDAR

^1^ Wearable sensors.

**Table 4:** Maximum concentrations for specific indoor air quality (IAQ) contaminants [99].

Parameter	Averaging Time	Limit for Acceptable IAQ	Unit
Particulate Matter ^1^	24 h	50	μg/m^3^
Ozone ^1^	8 h	120	μg/m^3^
Nitrogen Dioxide ^1^	1 h	200	μg/m^3^
Carbon Monoxide	8 h	10	mg/m^3^

^1^ Associated with the triggering of respiratory distress [97].

**Table 5:** List of machine learning classifiers used in the literature for ADL recognition.

Machine Learning Model	Classifiers	Reviewed Works
**Discriminative**	Support Vector Machine (SVM)	[62,67,69,70,127,128]
	Decision Tree	[99,100,101]
	Neural Networks	[96,97,98]
**Generative**	Hidden Markov Models (HMM)	[48,69,70,128]
	Naïve Bayes	[70,124]
